# Real-World Effectiveness and Renal Safety of Foscarnet for CMV Reactivation After Allogeneic Hematopoietic Stem Cell Transplantation

**DOI:** 10.3390/jcm15166365

**Published:** 2026-08-18

**Authors:** Leylagül Kaynar, Ibrahim Abdi Ali, Mahmoud Alrais, Amir Hossein Abedi, Süreyya Yiğit Kaya, Olgu Erkin Çınar, Hüseyin Saffet Beköz, Senem Maral

**Affiliations:** 1 Department of Hematology, Medipol Mega University Hospital, Istanbul Medipol University, 34810 Istanbul, Türkiye; sureyya.yigit@medipol.edu.tr (S.Y.K.); olgu.cinar@medipol.edu.tr (O.E.Ç.); hsbekoz@medipol.edu.tr (H.S.B.); senem.maral@medipol.edu.tr (S.M.); 2 Faculty of Medicine, Istanbul Medipol University, 34810 Istanbul, Türkiye; ibrahimabdi0600@gmail.com (I.A.A.); mahmoudsadeq2001@gmail.com (M.A.); abedimd@outlook.com (A.H.A.)

**Keywords:** cytomegalovirus infections, hematopoietic stem cell transplantation, foscarnet, acute kidney injury, nephrotoxicity

## Abstract

**Background/Objectives**: Cytomegalovirus (CMV) reactivation is a major cause of morbidity and mortality in allogeneic hematopoietic stem cell transplant (allo-HCT) recipients. Letermovir is used to prevent infections, but it is not available in many countries. Ganciclovir and valganciclovir carry a risk of bone marrow suppression. Foscarnet is frequently used as a bone marrow-sparing alternative; however, its clinical utility is constrained by nephrotoxicity. In this study, we aimed to evaluate the efficacy and renal safety of foscarnet and to identify factors associated with acute kidney injury (AKI) occurring during foscarnet treatment. **Methods**: This real-world, retrospective, single-center cohort study included 40 adult allo-HCT recipients treated with foscarnet for CMV reactivation between May 2022 and February 2025. **Results**: CMV polymerase chain reaction (PCR) negativity was achieved in 90% of patients, with a median time to PCR negativity of 10 (4–28) days. AKI occurred in 42.5% of patients, and 12.5% required hemodialysis. Foscarnet treatment was associated with a significant increase in serum creatinine levels and significant reductions in potassium, calcium, and magnesium concentrations (*p* < 0.05 for all). In univariable logistic regression analysis, myeloablative conditioning was associated with higher odds of AKI (OR 10.29, 95% CI 1.15–91.63; *p* = 0.037), while male sex showed numerically higher odds that did not reach conventional statistical significance (OR 4.28, 95% CI 0.96–19.01; *p* = 0.056). **Conclusions**: Among allo-HCT recipients treated with foscarnet, virological clearance was frequently observed, while AKI and electrolyte disturbances were common. These findings suggest that close renal and electrolyte monitoring may be warranted. However, given the retrospective, single-center design, small sample size, and absence of a comparator group, the findings should be interpreted cautiously and confirmed in larger prospective comparative studies.

## 1. Introduction

Cytomegalovirus (CMV) reactivation remains one of the most clinically significant infectious complications after allogeneic hematopoietic stem cell transplantation (allo-HCT), and continues to contribute to non-relapse morbidity and mortality despite modern monitoring strategies [1,2]. The risk factors for CMV infection include the serological status of both the donor and the recipient, the use of high-dose corticosteroids, T-cell depletion strategies, graft-versus-host disease (GVHD), and transplantation from donors other than Human Leukocyte Antigen (HLA)-matched related siblings [3]. In current transplant practice, the central challenge is balancing antiviral efficacy with treatment-related toxicity in highly immunocompromised patients [1,4].

Although letermovir prophylaxis has substantially reduced clinically significant CMV infection, access remains uneven across countries and centers [5]. Therefore, many centers still rely on preemptive treatment, where ganciclovir/valganciclovir are effective but limited by myelosuppression, especially during early engraftment [2,6]. In this setting, foscarnet is commonly selected to avoid additional bone marrow toxicity [6].

Foscarnet is a pyrophosphate analog that exerts its antiviral activity by reversibly inhibiting the viral deoxyribonucleic acid (DNA) polymerase pUL54 through blockade of the pyrophosphate-binding site [7]. Randomized and real-world studies show that foscarnet can provide meaningful virologic control after allo-HCT, but with a distinct renal and metabolic toxicity profile [2,6]. Importantly, long-term follow-up data suggest that foscarnet exposure may be associated with sustained renal function decline, highlighting the need for close renal monitoring and supportive care [4]. The incidence of foscarnet-associated renal toxicity following allo-HCT has been reported to range widely from 3% to 61.6%, depending on the definition of acute kidney injury (AKI), patient risk profile, and duration of therapy [2,4,8,9,10,11]. Notably, the risk increases substantially with prolonged treatment durations [4]. These data support evaluating outcomes beyond initial CMV polymerase chain reaction (PCR) clearance, including recurrence burden, AKI, dialysis requirement, and survival.

Despite the continued use of foscarnet in allo-HCT recipients, contemporary real-world evidence regarding its clinical outcomes remains limited. Previous studies have generally focused on virological response or nephrotoxicity separately, whereas data integrating virological clearance, CMV recurrence, renal and electrolyte abnormalities, and clinical factors associated with AKI during foscarnet treatment are scarce. This knowledge gap is particularly relevant because foscarnet is often selected in clinically complex patients with cytopenias, delayed engraftment, suspected ganciclovir resistance, or concomitant exposure to other nephrotoxic agents. Therefore, this retrospective, single-center study aimed to provide a descriptive real-world evaluation of virological response, CMV recurrence, renal and electrolyte abnormalities, and factors associated with AKI among allo-HCT recipients treated with foscarnet. The study was not designed to compare foscarnet with alternative antiviral treatments. Given the limited availability of letermovir prophylaxis in real-world settings and the scarcity of existing data on this subject, we sought to provide additional evidence regarding the clinical efficacy and nephrotoxicity profile of foscarnet.

## 2. Materials and Methods

### 2.1. Study Design and Setting

This retrospective, single-center observational cohort study was conducted at the Department of Hematology, Istanbul Medipol University Faculty of Medicine. The patients who underwent allo-HCT and received foscarnet for CMV reactivation between May 2022 and February 2025 were included. The objective of the study was to evaluate the real-world efficacy and renal safety of foscarnet for CMV reactivation after allo-HCT in a clinical setting where access to letermovir prophylaxis remains limited. This study was conducted in accordance with the Declaration of Helsinki and was approved by the Clinical Research Ethics Committee of Istanbul Medipol University (Approval number: E-10840098-202.3.02-4532, Date: 10 July 2025).

### 2.2. Patient Selection and Data Collection

Patients were eligible if they were ≥18 years, had undergone allo-HCT, had documented CMV reactivation, and received at least one dose of foscarnet. Demographic, transplant-related, virological, laboratory, treatment, and outcome data were extracted from electronic medical records. Baseline transplant variables included underlying diagnosis, donor type, conditioning intensity, anti-thymocyte globulin exposure, and post-transplant cyclophosphamide use. Conditioning regimens were categorized as myeloablative conditioning (MAC) or reduced-intensity conditioning (RIC), according to the criteria established by the Center for International Blood and Marrow Transplant Research [12]. CMV reactivation was defined as the detection of CMV DNA in plasma by quantitative PCR prompting initiation of preemptive antiviral therapy according to institutional monitoring protocols. Preemptive treatment followed a risk-adapted institutional approach. In HLA-matched sibling donor transplant recipients, treatment was generally initiated when CMV DNA exceeded 1000 IU/mL or when progressively increasing viral loads were observed on serial measurements despite remaining below this threshold. Recipients of haploidentical or mismatched donor grafts were considered at higher risk of CMV progression because of HLA disparity, more intensive immunosuppression, and potentially delayed CMV-specific immune reconstitution. Therefore, treatment could be initiated below 1000 IU/mL when serial PCR measurements demonstrated increasing DNAemia, particularly in the presence of GVHD or systemic corticosteroid exposure. No single lower numerical cutoff was applied; donor type was considered together with viral kinetics and the degree of immunosuppression.

### 2.3. CMV Monitoring and Definitions

Quantitative CMV DNA levels were measured using the cobas^®^ 6800 System with the cobas^®^ CMV assay (Roche Diagnostics, Basel, Switzerland), according to the manufacturer’s instructions. Results were reported in IU/mL with a lower limit of quantification of 34.5 IU/mL. Patients were monitored for CMV reactivation using quantitative CMV PCR testing twice weekly during the inpatient early post-transplant period [13]. Following discharge, CMV PCR was generally performed weekly, while higher-risk patients, including those with haploidentical or mismatched donor transplants, GVHD, corticosteroid exposure, or detectable CMV DNAemia, were monitored twice weekly according to institutional practice. The institutional CMV monitoring protocol remained unchanged throughout the study period. CMV infection and disease terminology followed contemporary consensus definitions where applicable [1].

Virological response was defined as two consecutive negative CMV PCR results after initiation of foscarnet therapy. CMV recurrence was defined as reappearance of detectable CMV DNAemia after prior PCR negativity during follow-up. CMV end-organ disease (e.g., CMV colitis) was recorded based on compatible clinical findings together with laboratory, endoscopic, and/or histopathologic evidence.

### 2.4. Foscarnet Treatment and CMV Management Approach

CMV surveillance is performed using serial quantitative PCR testing as part of routine post-transplant monitoring in our center. Preemptive antiviral therapy is initiated upon detection of clinically significant CMV DNAemia. Foscarnet was preferentially used in situations where avoidance of additional bone marrow suppression was clinically desirable, including early post-transplant periods, delayed engraftment, intolerance to ganciclovir or valganciclovir, or suspected antiviral resistance. Presumed ganciclovir resistance was defined as persistent or increasing CMV DNAemia despite at least 14 days of appropriately dosed intravenous ganciclovir therapy [1]. Routine CMV resistance genotyping (UL97/UL54 mutation analysis) was not available at our institution during the study period; therefore, the decision to switch to foscarnet was based on clinical and virological findings rather than molecular confirmation. Foscarnet was administered intravenously with dose adjustment according to renal function. Adequate hydration and close monitoring of renal function and electrolyte levels were routinely performed during therapy. The type and volume of hydration were individualized according to the patient’s clinical condition, renal function, and fluid tolerance, while the overall hydration strategy remained consistent throughout the study period. Serum potassium, calcium, magnesium, and phosphate levels were monitored daily during foscarnet therapy. Electrolyte abnormalities were corrected with oral and/or intravenous supplementation according to institutional practice.

Renal toxicity during foscarnet therapy was evaluated according to the Kidney Disease: Improving Global Outcomes (KDIGO) criteria [14]. AKI was defined as an increase in serum creatinine of ≥0.3 mg/dL within 48 h or ≥1.5 times the baseline value within 7 days. The requirement for hemodialysis during follow-up was recorded as a major renal outcome. Laboratory parameters, including serum creatinine, electrolytes, and hematologic indices, were recorded immediately before initiation of foscarnet therapy and on the last day of foscarnet treatment.

### 2.5. Statistical Analysis

The primary effectiveness outcome was CMV PCR negativity. Key safety outcomes were AKI and dialysis requirement. Survival outcomes included day-100 mortality, one-year mortality, non-relapse mortality (NRM), and overall survival (OS). The distribution of continuous variables was evaluated using the Kolmogorov–Smirnov test. Non-parametric data were compared using the Mann–Whitney U test, whereas parametric data was compared using Student’s *t*-test. The mean ± standard deviation was used to represent parametric variables, while the median (range) was used to express non-parametric variables. The categorical data were compared using the chi-square or Fisher’s exact tests. Changes in laboratory parameters before and after foscarnet therapy were evaluated using paired *t*-tests or Wilcoxon signed-rank tests, as appropriate.

The median follow-up duration was estimated using the reverse Kaplan–Meier method. Survival probabilities were estimated using the Kaplan–Meier method. OS was calculated from the initiation of foscarnet therapy to death from any cause or last follow-up. Potential factors associated with AKI during foscarnet therapy were evaluated using univariable logistic regression and reported as odds ratios (ORs) with 95% confidence intervals (CIs). Associations between baseline factors and mortality within 100 days after foscarnet initiation were explored using univariable Cox proportional hazards regression and reported as hazard ratios (HRs) with 95% CIs. Patients who remained alive were censored at the earlier of their last follow-up or day 100. Given the limited number of AKI events (*n* = 17) and deaths within 100 days (*n* = 6), multivariable models were not constructed because of the substantial risk of overfitting. Therefore, all regression analyses were considered exploratory. The data were analyzed using IBM SPSS Statistics for Windows, version 25.0 (IBM Corp., Armonk, NY, USA). A two-tailed *p*-value of less than 0.05 was considered statistically significant in all tests.

## 3. Results

### 3.1. Patient and Transplantation Characteristics

The median age was 40.5 (19–69) years, and 35% (*n* = 14) of patients were female. The most common diagnosis was acute leukemia in 24 (60%) patients. Regarding donor types, matched sibling donors were used in 50% (*n* = 20) of allo-HCT, matched unrelated donors in 25% (*n* = 10), haploidentical donors in 12.5% (*n* = 5), and mismatched unrelated donors in 12.5% (*n* = 5). MAC was administered in 75% (*n* = 30) of patients. Anti-thymocyte globulin was used in 40% (*n* = 16), and post-transplant cyclophosphamide in 55% (*n* = 22) (Table 1). The most frequently used conditioning regimens were fludarabine plus total body irradiation (*n* = 9, 22.5%) and fludarabine plus busulfan (*n* = 8, 20.0%), while the complete distribution of all conditioning regimens is provided in Supplementary Table S1.

### 3.2. Characteristics of CMV Reactivation and Foscarnet Therapy

The median time to foscarnet initiation after allo-HCT was 22 (7–180) days. The most common indication for foscarnet was avoidance of myelosuppression (75%), followed by suspected ganciclovir resistance (17.5%) and delayed engraftment (7.5%). Eighteen patients (45%) received primary CMV preemptive therapy before initiation of foscarnet therapy. The median baseline CMV viral load was 523 (72–18,872) IU/mL. The median duration of foscarnet therapy was 9 (2–28) days.

CMV-related clinical manifestations were observed in 23 (57.5%) patients, and CMV colitis was diagnosed in 7 (17.5%) patients. Virological clearance was achieved in 90% (*n* = 36) of patients, with a median time to PCR negativity of 10 (4–28) days. However, CMV recurrence occurred in 26 of the 36 patients who achieved virological clearance (72.2%). The median time to CMV recurrence after CMV PCR negativity was 22 (3–310) days (Table 2). Clinical outcomes were also evaluated according to the indication for foscarnet initiation. No significant differences were observed in treatment response, AKI incidence, dialysis requirement, or day-100 mortality according to the indication for foscarnet initiation (*p* > 0.05 for all, Supplementary Table S2).

### 3.3. Renal Toxicity and Laboratory Findings

**Figure 1:**
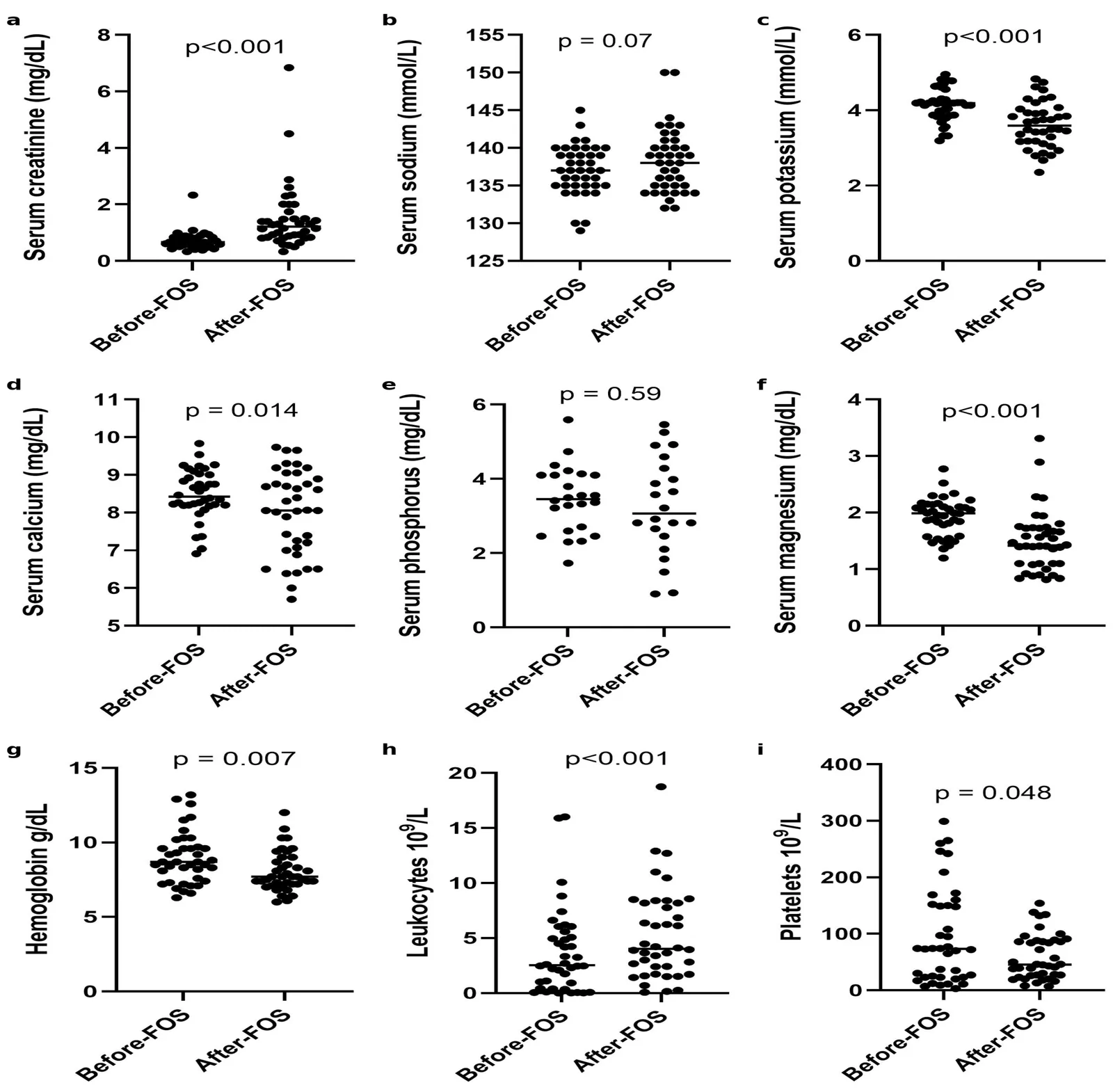
Changes in renal function, electrolyte levels, and hematologic parameters during foscarnet therapy. Scatter plots showing laboratory parameters measured before initiation and at the end of foscarnet therapy. (**a**) Serum creatinine, (**b**) sodium, (**c**) potassium, (**d**) calcium, (**e**) phosphorus, (**f**) magnesium, (**g**) hemoglobin, (**h**) leukocyte count, and (**i**) platelet count. FOS: foscarnet.

The median serum creatinine level increased from 0.66 (0.33–2.33) mg/dL at initiation of foscarnet therapy to 1.22 (0.33–6.84) mg/dL at the end of therapy (*p* < 0.001) (Figure 1a, Supplementary Table S3). During foscarnet therapy, AKI occurred in 17 patients (42.5%). Among these patients, 4 (23.5%) had KDIGO stage 1, 5 (29.4%) had stage 2, and 8 (47.1%) had stage 3 AKI. Five of the eight patients with stage 3 AKI required hemodialysis. Among electrolyte parameters, the median potassium level decreased from 4.19 (3.19–4.82) mmol/L before foscarnet therapy to 3.59 (0.9–5.46) mmol/L at the end of therapy (*p* < 0.001, Figure 1c). The median calcium and magnesium levels before foscarnet therapy were 8.42 (6.91–9.83) mg/dL and 2.02 (1.47–2.52) mg/dL, respectively, and decreased to 8.05 (5.7–9.73) mg/dL and 1.41 (0.84–3.31) mg/dL at the end of therapy (*p* = 0.014 and *p* < 0.001, respectively; Figure 1d,f). No statistically significant differences were observed in sodium and phosphorus levels before and after foscarnet therapy (Supplementary Table S3, Figure 1b,e).

Regarding hematologic parameters, significant changes were also observed following foscarnet therapy. The median hemoglobin level was 8.7 (6.6–13.2) g/dL before foscarnet therapy and 7.7 (6–12) g/dL after therapy (*p* = 0.007, Figure 1g). Platelet counts were lower at the end of foscarnet therapy compared with initiation, with median values of 73.5 (3–399) ×10^9^/L and 45.5 (7–154) ×10^9^/L, respectively (*p* = 0.048, Figure 1i). In contrast, the median leukocyte count increased from 2.98 (0.01–16.03) ×10^9^/L before foscarnet therapy to 4.03 (0.07–18.75) ×10^9^/L after therapy (*p* < 0.001, Figure 1h). Additional analyses were performed to evaluate potential confounding factors associated with AKI. No significant associations were observed between AKI and the presence of acute GVHD, hemodynamic instability, or concomitant exposure to nephrotoxic antimicrobial agents (all *p* > 0.05; Supplementary Table S4).

### 3.4. Survival Outcomes

The median follow-up duration, estimated using the reverse Kaplan–Meier method, was 16.3 months (95% CI: 10.7–21.9). The mean OS was 26.8 (95% CI: 21.9–31.7) months and the median survival was not reached. During follow-up, 11 patients (27.5%) died. Only one death was attributed to relapse. The most frequent cause of death was multi-organ failure (*n* = 4), followed by infection-related causes (*n* = 3), and GVHD (*n* = 3). The NRM rate was 25% (*n* = 10). The Kaplan–Meier estimates of OS at day 100 and one year were 84.7% and 72.4%, respectively. Crude mortality was 15.0% (6/40) by day 100 and 25.0% (10/40) by one year.

Potential prognostic factors associated with 100-day mortality were evaluated in univariate analysis (Table 3). Day-100 mortality occurred in 5 of 17 patients (29.4%) who developed AKI during foscarnet therapy versus 1 of 23 patients (4.3%) without AKI; in 3 of 5 patients (60.0%) who required hemodialysis versus 3 of 35 patients (8.6%) who did not; and in 5 of 14 patients (35.7%) admitted to the ICU versus 1 of 26 patients (3.8%) who were not admitted. Given the small number of deaths and because AKI, hemodialysis requirement, and ICU admission were post-baseline events whose timing was not incorporated into the analysis, these observations are presented descriptively, without formal comparative testing or survival modeling.

### 3.5. Factors Associated with Acute Kidney Injury

Factors associated with the development of AKI during foscarnet therapy were evaluated using logistic regression analysis (Table 4). In univariate analysis, myeloablative conditioning was associated with higher odds of AKI (OR 10.29, 95% CI 1.15–91.63; *p* = 0.037). Male sex also showed higher estimated odds of AKI, although the association did not reach conventional statistical significance (OR 4.28, 95% CI 0.96–19.01; *p* = 0.056).

## 4. Discussion

In this real-world, single-center cohort of allo-HCT recipients treated with foscarnet for CMV reactivation, we observed two significant findings: a high rate of initial virological clearance and a substantial burden of foscarnet-associated nephrotoxicity. CMV PCR negativity was achieved in 90% of patients, with a median time to clearance of 10 days; this demonstrates that foscarnet remains an effective treatment option for allo-HCT recipients where additional myelosuppression is undesirable. This is particularly important in the early post-transplantation period, when ganciclovir or valganciclovir may be avoided due to cytopenias, delayed graft engraftment, or pre-existing hematological toxicity [2,6,7]. In that regard, our findings support the continued role of foscarnet as an important bone marrow-sparing antiviral agent in centers where letermovir prophylaxis is not uniformly available or where breakthrough/reactivation episodes still require active treatment.

Foscarnet has demonstrated variable but generally favorable virological efficacy in the management of CMV infection after allo-HCT. In early randomized trials, foscarnet showed comparable efficacy to ganciclovir for prophylactic treatment after allo-HCT, while also offering a practical advantage such as avoiding serious bone marrow toxicity [2]. Similarly, Moretti et al. reported that foscarnet is a reasonable alternative to ganciclovir in the management of CMV antigenemia after allo-HSCT [6]. Metafuni et al. reported that overall virological response rates reached 87% in 97 patients, with a rapid mean time to viral clearance of 7 days, particularly in the preventive setting and in patients with lower baseline viral load [9]. In contrast, in the patient cohort with resistant or refractory CMV infection, virological clearance rates were lower, around 67%, and achieved more slowly; the mean time to response was 27 days [15]. Our study reported a 90% virological clearance rate and a median negativity duration of 10 days. Our results are comparably positive to prior real-world experiences and confirm that foscarnet maintains its antiviral activity in the allo-HCT setting. Although the initial virological response was strong, CMV recurrence occurred in 26 of the 36 patients (72.2%) who achieved initial virological clearance. This outcome most likely reflects the profound and prolonged immunosuppression characteristic of allo-HCT recipients, rather than any lack of antiviral efficacy from foscarnet. Factors such as delayed immune reconstitution, the need for corticosteroid therapy due to GVHD, and the absence of secondary prophylaxis with letermovir may all have contributed to this high rate of recurrence. Therefore, despite successful initial viral clearance, close CMV surveillance remains essential in this high-risk population.

Nephrotoxicity represents one of the major clinical limitations of foscarnet therapy [4,7]. Foster et al. emphasized that AKI rates were similar in allo-HCT patients using and not using foscarnet, but renal impairment at 12 months was significantly higher in foscarnet-treated patients [4]. In the randomized preemptive-therapy trial by Reusser et al., impaired renal function was reported in only 5% of foscarnet-treated patients with allo-HCT. However, in this study, patients with baseline creatinine clearance <60 mL/min were excluded, and the treatment duration was limited to a maximum of 4 weeks [2]. In the haploidentical or unrelated donor allo-HSCT cohort reported by Metafuni et al., renal failure was observed in 14% of patients, and creatinine doubling was seen in only 2.1% [9]. Inose et al. observed an AKI rate of 51.1%, but KDIGO-based criteria were used in this study [8]. In our study, AKI was defined using KDIGO criteria, and the AKI rate was 42.5%, with a hemodialysis requirement of 12.5%. The differences in nephrotoxicity rates observed in allo-HCT recipients using foscarnet are thought to be due to the significant heterogeneity seen in toxicity definitions.

In addition to its effects on renal function, foscarnet is well known to cause clinically significant electrolyte disturbances via tubular toxicity [7]. Despite a relatively low rate of clinically defined renal failure, hypocalcemia occurred in 22% of patients treated with foscarnet, hypomagnesemia in 18%, hypokalemia in 17% and hypophosphatemia in 6% [2]. Similarly, Avery et al. reported electrolyte disturbances including potassium, calcium, phosphate and magnesium in transplant recipients treated with foscarnet for resistant or refractory CMV infection [15]. In contrast, Inose et al. found that baseline hypokalemia, hypomagnesemia, and hypocalcemia were not significantly associated with subsequent AKI, suggesting that electrolyte abnormalities and creatinine-defined renal damage may be related but partially distinct manifestations of foscarnet toxicity [8]. Consistent with these findings, our study observed a significant decrease in potassium, calcium, and magnesium levels during foscarnet treatment; this supports the presence of clinically significant tubular dysfunction, which can occur with, but is not fully explained by, renal damage as defined by creatinine.

Despite the well-recognized nephrotoxicity of foscarnet, studies specifically investigating predictors of AKI occurring during foscarnet treatment remain scarce. Inose et al. demonstrated that foscarnet administration longer than 27 days was independently associated with a significantly increased incidence of AKI (OR 5.64, 95% CI: 1.32–24.2), highlighting the cumulative toxicity of prolonged exposure [8]. In contrast, no significant association between treatment duration and AKI was observed in our cohort. Instead, MAC was associated with significantly higher odds of AKI occurring during foscarnet treatment, while male sex showed a similar association that narrowly missed conventional statistical significance.

Although not consistently reported in studies specific to foscarnet, sex-related differences in AKI risk have been identified in the broader nephrology literature. Female sex is associated with relative protection, possibly through estrogen-related anti-inflammatory and endothelial effects, while male sex has been associated with increased susceptibility to both ischemic and nephrotoxic renal damage [16]. In addition, MAC may further contribute to renal vulnerability in this setting. Conditioning intensity is a well-known factor of organ toxicity following allo-HCT, with MAC carrying the highest risk of systemic and renal injury [17]. Moreover, MAC may further contribute to renal vulnerability, as higher-intensity conditioning regimens have been associated with increased regimen-related toxicity, including renal complications, likely reflecting cumulative exposure to nephrotoxic agents.

Foscarnet-associated nephrotoxicity represents not only a laboratory-defined adverse event but also a determinant of clinical outcome. Avery et al. reported that all allo-HCT recipients requiring renal replacement therapy during foscarnet therapy died, emphasizing the profound clinical impact of severe renal injury [15]. In our cohort, three of the five patients who required hemodialysis died by day 100. Although this finding suggests that dialysis-requiring AKI may identify a particularly high-risk clinical subgroup, it is based on very small numbers and should be considered descriptive and exploratory. Hemodialysis may reflect the severity of kidney injury and overall clinical deterioration rather than an independent or causal determinant of mortality.

This study has several limitations. Firstly, its single-center and retrospective design increases potential selection bias and restricts generalizability. Secondly, the small sample size and limited number of AKI events and day-100 deaths reduced statistical power and precision and precluded reliable multivariable modeling. Consequently, residual confounding cannot be excluded, and the wide confidence intervals indicate substantial imprecision; therefore, all observed associations should be considered exploratory and hypothesis-generating. Thirdly, AKI events were identified based on their occurrence during foscarnet treatment. However, the retrospective design did not permit formal causality adjudication or attribution of these events solely to foscarnet. In the post-transplant setting, multiple concomitant factors may have contributed to kidney injury, including conditioning regimen and intensity, prior antiviral exposure, calcineurin inhibitor use, infections, GVHD, hemodynamic instability, and exposure to other nephrotoxic agents. In addition, information regarding the presence or absence of tumor-associated autoimmune comorbidities was not systematically available, precluding assessment of their potential contribution to renal outcomes. Furthermore, variability in foscarnet dose, treatment duration, and supportive care strategies, including hydration procedures may have influenced both efficacy and toxicity results. Routine CMV resistance genotyping was unavailable during the study period; therefore, presumed ganciclovir resistance was defined according to clinical and virological criteria without molecular confirmation. Moreover, the availability of parameters such as resistance genotyping and immune system remodeling was inconsistent, which constrained a more thorough mechanistic explanation. Finally, the absence of a comparator group managed with alternative antiviral strategies precludes direct comparisons of the efficacy and safety of foscarnet with those of other approaches. Accordingly, our findings should be interpreted as descriptive real-world observations rather than evidence of the relative superiority or inferiority of foscarnet, and validation in larger multicenter cohorts is warranted.

## 5. Conclusions

In this real-world study, foscarnet has demonstrated its role as a bone marrow-sparing antiviral option by rapidly providing effective virological control of CMV reactivation in allo-HCT recipients; however, the risk of AKI remains significant and requires vigilant monitoring. In centers where letermovir prophylaxis is not available, foscarnet remains a critical therapeutic option. However, the use of foscarnet necessitates careful patient selection, rigorous monitoring of renal function and electrolytes, and the implementation of proactive supportive care measures. Future prospective studies should prioritize the development of risk-adapted strategies that carefully balance antiviral efficacy against toxicity, with the ultimate goal of enhancing early outcomes following transplantation.

## Figures and Tables

**Table 1:** Baseline Patient and Transplant Characteristics.

Characteristic	*n* (%)
Age (years) (median, range)	40.5 (19–69)
Sex (female)	14 (35)
Diagnosis	
Acute leukemia	24 (60)
Myelofibrosis	4 (10)
Hemoglobinopathies	3 (7.5)
Multiple myeloma	3 (7.5)
Lymphomas	3 (7.5)
CML/MDS	3 (7.5)
Comorbidities	10 (25)
Diabetes mellitus	6 (15)
Essential hypertension	3 (7.5)
Chronic obstructive pulmonary disease	3 (7.5)
Others	3 (7.5)
Donor Types	
Matched sibling donor	20 (50)
Matched Unrelated donor	10 (25)
Haploidentical donor	5 (12.5)
9/10 mismatched unrelated donor	5 (12.5)
Conditioning Regimens	
MAC	30 (75)
RIC/NMA	10 (25)
Anti-thymocyte globulin	16 (40)
Post-transplant cyclophosphamide	22 (55)

CML, chronic myeloid leukemia; MDS, myelodysplastic syndrome; MAC, myeloablative conditioning; RIC, reduced-intensity conditioning; NMA, non-myeloablative conditioning.

**Table 2:** Characteristics of CMV infection and foscarnet therapy.

Parameter	*n* (%) or Median (Range)
Primary CMV preemptive therapy before foscarnet	18 (45)
Duration of CMV preemptive therapy before foscarnet (days)	8.5 (3–29)
Baseline CMV viral load (IU/mL)	523 (72–18,872)
Clinical symptoms of CMV infection	23 (57.5)
Concurrent CMV colitis	7 (17.5)
Indication for foscarnet therapy	
Alternative treatment to avoid bone marrow suppression	30 (75)
Suspected ganciclovir resistance	7 (17.5)
Delayed engraftment	3 (7.5)
Time to foscarnet initiation after transplantation (days)	22 (7–180)
Initial foscarnet dose of 180 mg/kg/day	23 (57.5)
Foscarnet dosing frequency—Twice daily	32 (80)
Foscarnet dosing frequency—Three times daily	8 (20)
Duration of foscarnet therapy (days)	9 (2–28)
Dose reduction required	8 (20)
CMV virological response (PCR negativity achieved)	36 (90)
Time to CMV PCR negativity (days)	10 (4–28)
CMV recurrence after foscarnet therapy	26/36 (72.2)
Time to CMV recurrence after PCR negativity (days)	22 (3–310)
Acute graft-versus-host disease (GVHD)	16 (40)
Skin GVHD	16 (40)
Gastrointestinal GVHD	10 (25)
Liver GVHD	3 (7.5)
Pulmonary GVHD	1 (2.5)
Day-100 mortality	6 (15)
One-year mortality	10 (25)

CMV: cytomegalovirus, PCR: polymerase chain reaction, GVHD: graft-versus-host disease.

**Table 3:** Exploratory univariable analyses of factors associated with day-100 mortality.

			Univariable Model		
Parameter	Compared Factors	Deaths/Total	HR	95% CI	*p*
Age (years)	>40.5 vs. ≤40.5	3/20 vs. 3/20	0.96	0.19–4.74	0.96
Sex	male vs. female	5/26 vs. 1/14	2.82	0.33–24.1	0.35
Diagnosis	Other diagnoses vs. leukemia	2/16 vs. 4/24	0.68	0.13–3.73	0.66
Comorbidity	present vs. absent	2/10 vs. 4/30	1.51	0.28–8.24	0.63
Donor	Haploidentical vs. other donor types	2/5 vs. 4/35	4.44	0.81–24.36	0.09
PTCy	Yes vs. no	5/22 vs. 1/18	4.39	0.51–37.55	0.18
TBI	Yes vs. no	4/16 vs. 2/24	3.24	0.59–17.71	0.18
Baseline CMV viral load (IU/mL)	>523 vs. ≤523	1/20 vs. 5/20	0.18	0.02–1.55	0.12

CMV, cytomegalovirus; TBI, total body irradiation; PTCy, post-transplant cyclophosphamide.

**Table 4:** Exploratory univariable analyses of factors associated with acute kidney injury.

			Univariable Model		
Parameter	Compared Factors	Events/Total	OR	95% CI	*p*
Age (years)	>40.5 vs. ≤40.5	10/20 vs. 7/20	1.86	0.52–6.61	0.34
Baseline serum creatinine (mg/dL)	>0.66 vs. ≤0.66	11/19 vs. 6/21	3.44	0.92–12.79	0.07
Sex	male vs. female	14/26 vs. 3/14	4.28	0.96–19	0.056
Diagnosis	Other diagnoses vs. leukemia	8/16 vs. 9/24	1.67	0.46–6.01	0.44
Comorbidity	present vs. absent	5/10 vs. 12/30	1.50	0.36–6.32	0.58
Donor	haploidentical vs. other donor types	4/5 vs. 13/35	6.77	0.68–67.25	0.1
Conditioning regimen	MAC vs. RIC	16/30 vs. 1/10	10.29	1.15–91.63	0.037
PTCy	Yes vs. no	9/22 vs. 8/18	0.87	0.25–3.05	0.82
TBI	Yes vs. no	7/16 vs. 10/24	1.09	0.3–3.91	0.89
Acute GVHD	Yes vs. no	7/16 vs. 10/24	0.92	0.29–2.95	0.89
Baseline CMV viral load (IU/mL)	>523 vs. ≤523	8/20 vs. 9/20	0.82	0.23–2.86	0.75
CMV end-organ disease	Yes vs. no	4/7 vs. 13/33	2.05	0.39–10.7	0.39
BK viremia	Yes vs. no	7/14 vs. 10/26	1.6	0.43–5.94	0.48
Time to foscarnet initiation after transplantation (days)	> 22 vs. ≤ 22	9/19 vs. 8/21	1.46	0.42–5.15	0.55
Treatment duration (days)	>9 vs. ≤ 9	7/18 vs. 10/22	0.76	0.21–2.71	0.68
Foscarnet dosing frequency	three times daily vs. twice daily	3/8 vs. 14/32	0.77	0.16–3.79	0.75

BK, BK polyomavirus; CMV, cytomegalovirus; GVHD, graft-versus-host disease; TBI, total body irradiation; PTCy, post-transplant cyclophosphamide; MAC, myeloablative conditioning; RIC, reduced-intensity conditioning.

## Data Availability

The data presented in this study are available upon reasonable request from the corresponding author. The data are not publicly available due to privacy and ethical restrictions.

## Supplementary Materials

The following supporting information can be downloaded at: https://www.mdpi.com/article/10.3390/jcm15166365/s1, Table S1: Distribution of conditioning regimens before allogeneic hematopoietic stem cell transplantation, Table S2: Clinical outcomes according to the indication for foscarnet initiation, Table S3: Laboratory parameters before and after foscarnet therapy, Table S4: Potential confounding factors associated with acute kidney injury.

## Abbreviations

The following abbreviations are used in this manuscript:

AKI	Acute Kidney Injury
allo-HCT	Allogeneic Hematopoietic Stem Cell Transplantation
ATG	Anti-Thymocyte Globulin
BK	BK Polyomavirus
CI	Confidence Interval
CMV	Cytomegalovirus
DNA	Deoxyribonucleic acid
GVHD	Graft-versus-Host Disease
HLA	Human Leukocyte Antigen
HR	Hazard Ratio
ICU	Intensive Care Unit
KDIGO	Kidney Disease: Improving Global Outcomes
MAC	Myeloablative Conditioning
NMA	Non-Myeloablative Conditioning
NRM	Non-Relapse Mortality
OR	Odds Ratio
OS	Overall Survival
PCR	Polymerase Chain Reaction
PTCy	Post-Transplant Cyclophosphamide
RIC	Reduced-Intensity Conditioning
