# Supplementary material for: Real-World Effectiveness and Renal Safety of Foscarnet for CMV Reactivation After Allogeneic Hematopoietic Stem Cell Transplantation
Source: J Clin Med. 2026 Aug 18;15(16):6365. doi: 10.3390/jcm15166365 (PMC13513787; doi:10.3390/jcm15166365)
Supplement: Supplementary file 1 [file jcm-15-06365-s001.zip › jcm-4471516-supplementary.pdf]

Supplementary Table S1. Distribution of conditioning regimens before allogeneic hematopoietic stem cell transplantation

**Conditioning regimen**	**n**	**%**
Fludarabine + Total Body Irradiation (Flu/TBI)	9	22.5
Fludarabine + Busulfan (Flu/Bu)	8	20.0
Cyclophosphamide + Total Body Irradiation (Cy/TBI)	5	12.5
Treosulfan + Fludarabine + Thiotepa	3	7.5
Treosulfan + Fludarabine	3	7.5
Fludarabine + Busulfan + Thiotepa	2	5.0
Melphalan	2	5.0
Busulfan + Cyclophosphamide	1	2.5
Busulfan + Cyclophosphamide + Fludarabine	1	2.5
Total Body Irradiation alone	1	2.5
ATG + Thiotepa + Fludarabine	1	2.5

Supplemantary Table S2. Clinical outcomes according to the indication for foscarnet initiation

Outcome	Avoidance of engraftment delay (n = 3)	Suspected ganciclovir resistance (n = 7)	Avoidance of myelosuppression (n = 30)	*p* value
**Treatment response**, n (%)	3 (100.0)	5 (71.4)	28 (93.3)	0.256
**Acute kidney injury (AKI)**, n (%)	2 (66.7)	3 (42.9)	12 (40.0)	0.755
**Dialysis requirement**, n (%)	1 (33.3)	2 (28.6)	2 (6.7)	0.108
**Day-100 mortality**, n (%)	0 (0.0)	1 (14.3)	5 (16.7)	1.000

Supplemantary Table S3. Laboratory parameters before and after foscarnet therapy

Parameter	Before Foscarnet (median, range)	After Foscarnet (median, range)	P value
Serum creatinine (mg/dL)	0.66 (0.33 – 2.33)	1.22 (0.33 – 6.84)	<0.001
Sodium (mmol/L)	138 (130 – 145)	138.5 (132 – 150)	0.07
Potassium (mmol/L)	4.19 (3.19 – 4.82)	3.59 (0.9 – 5.46)	< 0.001
Calcium (mg/dL)	8.42 (6.91 - 9.83)	8.05 (5.7 – 9.73)	0.014
Phosphorus (mg/dL)	3.39 (1.73 – 5.59)	3.06 (0.9 – 5.46)	0.59
Magnesium (mg/dL)	2.02 (1.47 – 2.52)	1.41 (0.84 – 3.31)	< 0.001
Hemoglobin (g/dL)	8.7 (6.6 – 13.2)	7.7 (6 – 12)	0.007
Leukocytes ($\times 10^9$/L)	2.98 (0.01 – 16.03)	4.03 (0.07 – 18.75)	< 0.001
Platelets ($\times 10^9$/L)	73.5 (3 – 399)	45.5 (7 – 154)	0.048

Supplementary Table S4. Potential confounding factors associated with acute kidney injury

Variable	AKI (-) (n=23)	AKI (+) (n=17)	*p* value
Acute GVHD*, n (%)	9 (39.1)	7 (41.2)	1.000
Hemodynamic instability, n (%)	7 (30.4)	7 (41.2)	0.521
Concomitant nephrotoxic antimicrobial exposure†, n (%)	5 (21.7)	5 (29.4)	0.717

* Acute GVHD was predominantly mild (grade 1–2). Only two patients had severe acute GVHD (one grade 3 and one grade 4), precluding meaningful statistical analyses according to GVHD severity.

† Concomitant nephrotoxic antimicrobial exposure was defined as treatment with one or more of the following agents during foscarnet therapy: intravenous vancomycin (n = 1), teicoplanin (n = 5), aminoglycosides (n = 3), polymyxins (n = 3), and liposomal amphotericin B (n = 1). All patients received cyclosporine-based GVHD prophylaxis. Therefore, comparative analyses according to calcineurin inhibitor exposure were not feasible.
